# Leih- und Zeitarbeit in der Intensivpflege: Auswirkungen auf die Patientenversorgung der Überwachungs- und Intensivstationen

**DOI:** 10.1007/s00063-020-00753-5

**Published:** 2020-10-21

**Authors:** C. Hermes, C. Petersen-Ewert

**Affiliations:** 1 Friedrich-Ebert-Straße 60, 53177 Bonn, Deutschland; 2 Fakultät Wirtschaft & Soziales – Department Pflege & Management, Hochschule für Angewandte Wissenschaften Hamburg (HAW Hamburg), Hamburg, Deutschland

**Keywords:** Patienten Outcome, Arbeitnehmerüberlassung, Leiharbeiter, Qualität der Gesundheitsversorgung, Arbeitsbedinungen, Patient outcome, Agency worker, Temporary staff, Quality of health care, Working conditions

## Abstract

**Hintergrund:**

Leiharbeiter werden, meist im Sinne einer Arbeitnehmerüberlassung, zur pflegerischen Versorgung von Intensivpatienten eingesetzt. Ob bzw. wie sich Leiharbeit in der Pflege auf die Patientenversorgung auswirkt, wurde bislang kaum untersucht.

**Ziel:**

Zweck dieser systematischen Übersichtsarbeit ist es, die verfügbaren Forschungsergebnisse über den Einsatz von Leiharbeitern in der pflegerischen Versorgung auf Intensiv- und Überwachungsstationen zu beschreiben und die potenziellen Auswirkungen auf das Patientenoutcome zusammenzufassen.

**Methode:**

Es wurde in sieben Datenbanken mit booleschen Operatoren systematisch nach englisch- und deutschsprachigen Studien recherchiert und in Anlehnung an das PRISMA-Schema ausgewertet. Referenzen der Studien wurden ebenfalls in die Suche inkludiert und die Qualität aller eingeschlossenen Studien nach Hawker-Kriterien bewertet.

**Ergebnis:**

Von insgesamt *N* = 630 gesichteten Datensätzen konnten jeweils eine qualitative und zwei quantitative Studien identifiziert und in die Auswertung einbezogen werden. Die Ergebnisse der qualitativen Studien gaben nicht signifikant an, dass Leiharbeiter zu einem schlechteren Patientenoutcome beitragen können. Die Ergebnisse der quantitativen Studien zeigten, dass die Wahrscheinlichkeit für das Auftreten von katheterassoziierten Infektionen mit dem Einsatz von Leiharbeitern steigen kann, aber eher von der Stationsgröße anhängig ist: Je zusätzlichem Bett steigt die Wahrscheinlichkeit für eine VAP um 14,8 % (95 %-CI = 1,032–1,277, *p* = 0,011). Allerdings konnten Tendenzen für einen Rückgang der Sepsisrate, sobald weniger Leiharbeiter (Stunden/Patient) auf der Intensivstation eingesetzt wurden, nicht bestätigt werden.

**Schlussfolgerung:**

In den wenigen auswertbaren Studien wurden keine Hinweise dafür gefunden, dass der Einsatz von Leiharbeitern auf Intensiv- (ITS) und Überwachungsstationen (IMC) einen signifikanten Einfluss auf das Patientenoutcome hat. Es wurden allerdings Hinweise gefunden, dass individuelle Qualifikationen und die Arbeitsbedingungen einen Einfluss auf das Outcome haben. Weitere Studien sollten betrachten, welches Verhältnis von Festangestellten zu Leiharbeitern als unkritisch anzusehen ist, welche Qualifikationen temporäre Mitarbeiter vorweisen sollten und inwieweit diese überprüft werden können.

**Zusatzmaterial online:**

Die Online-Version dieses Beitrags (10.1007/s00063-020-00753-5) enthält* die vollständige Suchstrategie je Datenbank*. Beitrag und Zusatzmaterial stehen Ihnen auf www.springermedizin.de zur Verfügung. Bitte geben Sie dort den Beitragstitel in die Suche ein, das Zusatzmaterial finden Sie beim Beitrag unter „Ergänzende Inhalte“.

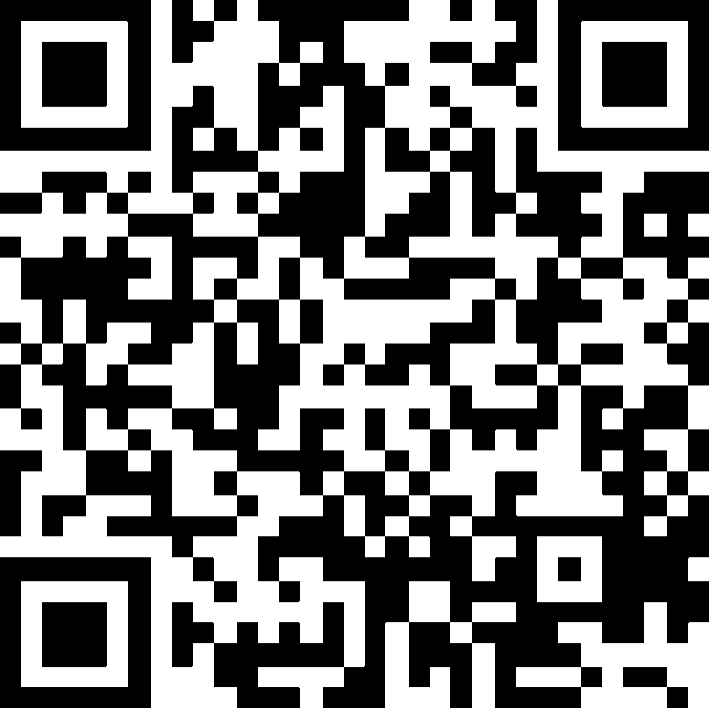

## Abstract (en)

**Background:**

Temporary contract workers are used for the nursing care of intensive care patients, usually by service providers in the sense of temporary employment. If or how temporary contract work has an impact on patient care has scarcely been investigated so far.

**Aim:**

The aim of this systematic review is to describe the available research results on the use of temporary workers in nursing care in intensive and intermediate care units and to summarize the prospective effects on patient outcomes.

**Method:**

Seven databases were systematically searched for English and German language articles using Boolean operators and evaluated according to the PRISMA schema. References of included studies were included in the search and the quality of all included studies was evaluated according to the Hawker criteria.

**Result:**

From a total of 630 datasets viewed, 1 qualitative and 2 quantitative studies were identified and analyzed. The findings of the quantitative studies indicated that the probability of the occurrence of catheter-associated infections can increase with the use of temporary workers, but is more dependent on the size of the unit: For each additional bed, the probability of VAP increases by 14.8% (95%-CI = 1.032–1.277, *p* = 0.011). However, trends for a decrease in the sepsis rate as soon as fewer temporary workers (hours/patient) were deployed in the intensive care unit (ICU) could not be confirmed.

**Conclusion:**

In the few available studies no evidence was found that the use of temporary workers in intensive care units (ICU) and intermediate care units (IMC) has a significant impact on patient outcomes; however, evidence was found that individual qualifications and working conditions have an influence on outcomes. Further studies should consider what ratio of permanent to temporary workers should be considered uncritical, what qualifications temporary workers should have and to what extent these can be checked.

## Einleitung

Seit 1991 sind die Behandlungsfälle in deutschen Kliniken um 25 % gestiegen, während gleichzeitig die Anzahl der Krankenhäuser von 2400 auf 1942 gesunken ist [1]. Hieraus lässt sich eine deutliche Arbeitsverdichtung ableiten, die durch den demografischen Wandel verstärkt wird. In den nächsten 20 Jahren werden in Deutschland ein weiterer Rückgang der Bevölkerung im Erwerbsalter und ein Anstieg der Seniorenzahl zu verzeichnen sein [2]. Gesundheitsorganisationen müssen ökonomisch agieren und stehen unter einem zunehmenden Druck, ihre Kosten zu senken [3]. Auf der Ausgabenseite sind die Personalkosten der größte Kostenfaktor im Krankenhaus. Im Gegensatz zu den ärztlichen Leistungen werden die pflegerischen Leistungen im deutschen DRG-System nur unzureichend auf der Erlösseite abgebildet [4]. Hieraus begründet wurden vor allem im pflegerischen Bereich in den letzten Jahren massive Personaleinsparungen getätigt. Eine Folge sind ein zunehmender Personalmangel auf der Intensivstation (ITS) und Intermediate-Care-Stationen (IMC; [5]). Obwohl es in Deutschland, verglichen mit allen anderen europäischen Ländern, überdurchschnittlich viele Intensiv- und Intermediate-Care-Betten bezogen auf die Einwohnerzahl gibt (etwa 30 Betten pro 100.000 Einwohner; [6]), kommt es paradoxerweise in diesem Bereich immer wieder zu Engpässen in der Patientenversorgung. Diese Engpässe sind maßgeblich darauf zurückzuführen, dass sowohl die Anzahl der Intensivbetten als auch die Behandlungsfälle in Deutschland kontinuierlich gestiegen sind, während im selben Zeitraum die Anzahl an Pflegekräften und Fachpflegekräften abgenommen hat [7]. Eine geringe Personalstärke und ein geringer Qualifikationsmix erhöhen die Wahrscheinlichkeit, dass Patienten im Krankenhaus sterben [8]. Mittlerweile ist die Anzahl der Pflegekräfte auf deutschen Intensivstationen nicht mehr ausreichend, um eine kontinuierliche und adäquate Patientenversorgung sicherzustellen. In der Folge müssen Betten und Behandlungsplätze in der Regel- und Notfallversorgung gesperrt werden [5]. Mit verschiedenen Mitteln wird versucht, den akuten Personalmangel auszugleichen [9]. Krankenhausmanager haben seit mehr als 15 Jahren zunehmend Schwierigkeiten, Pflegepersonal zu rekrutieren und/oder zu halten. Dabei wird auf entsprechende Vermittlerfirmen zurückgegriffen, um freie Stellen mit Leiharbeitern zu besetzen und eine adäquate Patientenversorgung aufrechtzuerhalten [10]. Eine Umfrage im Namen der Deutschen Gesellschaft für Internistische Intensiv- und Notfallmedizin (DGIIN) zeigte, dass trotz aller dieser Bemühungen der letzten Jahre 37 % der befragten Intensivpflegenden planen, ihren Beruf in den nächsten fünf Jahren zu verlassen. Zudem wollen insgesamt 34 % ihre Arbeitszeit in den nächsten zwei Jahren reduzieren. Nicht wenige Pflegekräfte scheinen dabei momentan einen Wechsel in die Leih- und Zeitarbeit zu bevorzugen [11].

Die Begriffe Leih- und Zeitarbeiter werden meist synonym verwendet und bezeichnen Mitarbeiter, die zeitlich begrenzt durch einen entsprechenden Personaldienstleister (Arbeitnehmerüberlassung) vermittelt werden. In der internationalen Literatur werden ebenfalls verschiedene Begriffe zur Leih- und Zeitarbeit synonym verwendet. Die Vermittlung durch eine Agentur ist auch in anderen Ländern üblich. Eine reine freiberufliche und/oder selbstständige Tätigkeit als Pflegekraft ist in deutschen Kliniken durch das aktuelle Urteil des Bundessozialgerichts (Az. BSG B 12 R 11/18 R) nicht mehr möglich. Für diesen Text wird im Folgenden der Begriff Leiharbeiter für alle Formen der Leih- und Zeitarbeit und Arbeitnehmerüberlassung verwendet.

Für die Intensivstation ist beschrieben, dass der Betreuungsschlüssel die sogenannten pflegesensitiven Outcomeparameter beeinflusst [12, 13]. Für die Normalstationen gibt es Hinweise, dass eine Versorgung mit mehr als 1,5 h pro Patient und Tag durch Zeitarbeiter mit einem erhöhten Sterberisiko verbunden ist [14]. Eine Ursache könnte fehlende Vertrautheit mit den Stationsabläufen und den internen Kommunikationsabläufen sein [15, 16]. Ob dies auch auf Intensivstationen zutrifft, ist unklar.

## Zielsetzung

Die Leiharbeit hat für den Gesundheitssektor in den letzten Jahren große Bedeutung erlangt. Es ist anzunehmen, dass dadurch die Personalstruktur von Stationen zunehmend verändert wird. Hierbei stellt sich die Frage, ob der Einsatz von Leiharbeitern das Patientenoutcome beeinflusst. Ziel dieser systematischen Literaturübersicht ist es, mögliche Zusammenhänge zwischen Leiharbeitern auf Intensiv- und Überwachungsstationen und Mortalität, Liegedauer, Hygienefehlern, Infektionen, Dekubitalulzera, körperlichen Fixierungen, Pflegezeit, Herz-Kreislauf-Ereignissen sowie allgemeiner Patientengefährdung und Patientensicherheit zu untersuchen.

## Methode

### Design

**Figure 1:**
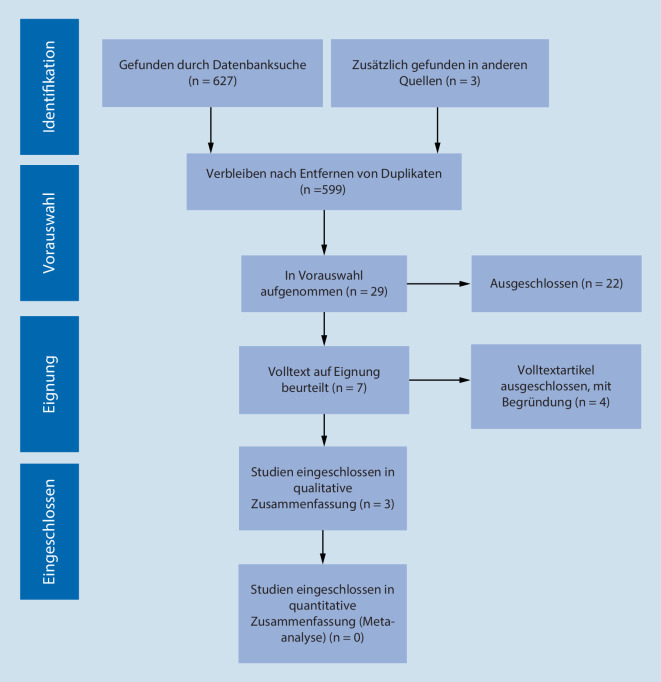

Die systematische Literaturrecherche wurde in Anlehnung an den PRISMA-Richtlinien (Preferred Reporting Items for Systematic Reviews and Meta-Analyses) durchgeführt (Abb. 1; [17]). Die Suche wurde vorab nicht registriert. Das PRISMA-Protokoll kann beim Erstautor angefordert werden.

### Suchstrategie

Es wurde in sieben elektronischen Datenbanken (1. PubMed, 2. CINAHL (Cumulative Index to Nursing and Allied Health Literature), 3. LIVIVO inkl. BASE (Bielefeld Academic Search Engine), 4. Wiley, 5. MEDLINE via PRIME, 6. Trip Medical Database und 7. Cochrane Library) zwischen Dezember 2019 und Ende März 2020 systematisch recherchiert. Eine Überprüfung auf neue Einträge erfolgte zuletzt am 16. April 2020. Um Studien zu identifizieren, die den Zusammenhang zwischen dem Einsatz von Leiharbeitern und einen Einfluss auf die Mortalität oder andere pflegesensitive Outcomeparameter untersuchten, wurde in 4 aufeinander folgenden Schritten vorgegangen:

Im ersten Schritt wurde die Fragestellung „Hat der Einsatz von Leiharbeitern auf der Intensiv- und Überwachungsstation gegenüber dem Einsatz von festangestellten Mitarbeitern einen Einfluss auf das Patientenoutcome?“ mittels PICO-Schema (Tab. 1) operationalisiert. Es folgte eine selektive Freitextsuche in der Suchmaschine Google Scholar zur zentralen Bestimmung der notwenigen Begrifflichkeiten. Als zweiter Schritt erfolgte die systematische Abfrage der Begriffe als MeSH-Begriffe, Schlüsselwörter und/oder kontrolliertes Vokabular in jeder der genannten Datenbanken mit booleschen Operatoren und Trunkierungen. Als unabhängige Variablen wurden die Begriffe der Leih- und Zeitarbeit und als abhängige Variablen die unerwünschten Ereignisse für Patienten definiert. Hierunter fielen die Parameter Mortalität, Liegedauer, Patientensicherheit, Hygienefehler, Infektionen, Dekubitalulzera, körperliche Fixierungen, Pflegezeit, Herz-Kreislauf- und allgemeine Patientengefährdungen. Eingegrenzt wurde die Suche auf den Bereich der kritisch kranken Patienten auf Überwachungs- und Intensivstationen. Die folgenden Begriffe wurden berücksichtigt: „agency work*“ OR „contract work“ OR „Temp* Staff*“ OR „Temporary Staff*“ OR „temporary nursing staff“ OR „agency nursing“ OR „Agency staff“ OR „Contract worker“ OR „Travel Nurse“ OR „self-employed nurse“ OR „temp nurse“ AND „patient outcome“ OR „patient outcomes“ OR „pressure ulcers“ OR „nursing time“ OR Restraint OR „physical restraints“ OR „nursing hours“ OR Hygiene OR Failure-to-rescue OR „cardiac events“ OR „patient safety“ OR „adverse events“ OR Infections OR „Length of stay“ OR „Health* provision“ OR „Mortality“ AND „ICU“ OR „critical care“ OR „critical care*“ OR intensive care unit. Die vollständige Suche je Datenbank findet sich im elektronischen Zusatzmaterial online.

**Table 1:** 

*P*atient/*P*opulation	Stationäre Patienten auf Intensivstation und/oder Überwachungsstation
*I*ntervention	Pflegekräfte auf Basis Leih- und Zeitarbeit
*„C*omparison“	Pflegekräfte auf Basis Festanstellung
*O*utcome	Mortalität, Liegedauer, Hygienefehler, Infektionen, Dekubitalulzera, körperliche Fixierungen, Pflegezeit, Herz-Kreislauf-Ereignisse sowie allgemeine Patientengefährdungen und Patientensicherheit

Im dritten Schritt wurden die Titel und Abstracts der Studien und bei Bedarf der vollständige Text gesichtet, um zu entscheiden, ob diese in die Auswertung aufgenommen werden.

### Einschlusskriterien

Studien wurden einbezogen, wenn diese 1) in Englisch oder Deutsch verfasst waren, 2) auf einem quantitativen oder qualitativen Forschungsdesign basierten, 3) den Zusammenhang zwischen Leih- und Zeitarbeitern und mindestens einem gesuchten Outcomeparameter berichteten, 4) in einem klinisch stationären Akutversorgungsumfeld für Erwachsene, wie Intensiv- und Überwachungsstationen, durchgeführt und 5) in einer von Fachkollegen begutachteten Zeitschrift (Peer-Review-Journal) veröffentlicht wurden. Ausgeschlossen wurden Studien, die 1) andere Settings als stationäre Akutkrankenhäuser beschrieben (z. B. Pflegeheime, Rehabilitations- und Weaning-Stationen, häusliche und ambulante Pflege), 2) nur über Kostenmodelle ohne Patientenoutcome berichteten, 3) zu mehr als 50 % pädiatrische Patienten betrachteten, 4) keine Studien waren (wie z. B. Stellungnahmen auf Grundlage von Wahrnehmungen, Letter und/oder Editorials) oder 5) im Recherchezeitraum und Überprüfungsstichtag nur als Abstract und/oder „corrected proofs“^1^ zur Verfügung standen.

Um weitere Studien zu identifizieren, die ebenfalls die Einschlusskriterien erfüllten, wurde die Suche im vierten Schritt mit einer Sichtung und Bewertung der Referenzlisten der eingeschlossenen Volltexte abgeschlossen.

### Qualitätssicherung

Zur Vorbereitung der inhaltlichen Auswertung wurden alle hierfür identifizierten Volltexte mittels eines spezifischen Bewertungsbogens nach Hawker et al. [18] bewertet (Tab. 2). Der Bewertungsbogen setzt sich aus neun Kategorien zusammen:

**Table 2:** 

Autoren	Titel und Abstract	Einleitung und Ziele	Methode und Daten	Stichprobe	Datenanalyse	Ethik und Bias	Ergebnisse	Übertragbarkeit und Generalisierbarkeit	Implikation und Nutzen	Summe
Bae SH, Brewer CS, Kelly M, Spencer A [19]	4	4	4	4	4	3	4	3	3	33
Shuldham C, Parkin C, Firouzi A, Roughton M, Lau-Walker M [20]	4	4	4	2	4	4	4	3	3	32
Hass H, Coyer FM, Theobald KA [21]	4	3	4	2	4	1	4	3	4	29
Fugulin FM, Rossetti AC, Ricardo CM, Possari JF, Mello MC, Gaidzinski RR [22]	2	2	2	1	2	2	3	2	2	18
Garcia PC, Fugulin FM [23]	2	2	2	1	2	2	3	2	2	18
Mazurenko O, Liu D, Perna C [24]	1	1	2	1	1	1	2	1	2	12
Hart P and Davis N [13]	2	3	3	3	3	2	3	3	3	25

1) Zusammenfassung und Titel, 2) Einleitung und Ziel(e), 3) Methode und Daten, 4) Stichprobe, 5) Datenanalyse, 6) Ethik und Bias, 7) Ergebnisse, 8) Übertragbarkeit und Generalisierbarkeit und 9) Implikationen und Nutzen. Diese Kategorien werden mit 1–4 (4 = gut, 3 = in Ordnung, 2 = mangelhaft, 1 = sehr mangelhaft) mittels spezifischer Fragestellungen bewertet. In die Datenanalysen wurden Arbeiten eingeschlossen, die mindestens 75 % (entspricht mindestens 27 von 36 Punkten) der möglichen Gesamtpunktzahl erreichten.

## Ergebnisse

Insgesamt wurden *n* = 627 Datensätze durch die systematische Suche in den Datenbanken identifiziert und *n* = 3 Studien aus der Referenzliste der eingeschlossen Volltexte händisch hinzugefügt (Abb. 1). Aus den *n* = 627 Studien der Datenbanken (Wiley *n* = 103; CINAHL *n* = 60; PubMed *n* = 18; LIVIVO *n* = 358; PRIME *n* = 11; Trip *n* = 74; Cochrane *n* = 3) wurden *n* = 31 Doppelungen entfernt. Bei *n* = 599 Datensätzen wurde zunächst ein Screening von Titel und Abstract zur Identifikation möglicher Volltexte durchgeführt. Ein Großteil der Ergebnisse aus den Datenbanken LIVIVO und Trip wurde aussortiert, da es sich um Verweise auf im Internet hinterlegte Präsentationen handelte und/oder Quellen nicht überprüfbar waren. Insgesamt konnten nach der Sichtung von Titel und Abstract *n* = 29 mögliche Volltexte identifiziert werden, wovon *n* = 22 ausgeschlossen wurden. Die Gründe waren:

1. Es wurde kein nachvollziehbarer Zusammenhang zwischen ausgewählten Variablen hergestellt (*n* = 8).
2. Es wurde nur über Kosten ohne Zusammenhang zum Patientenoutcome berichtet (*n* = 4).
3. Die erhobenen Daten unterschieden nicht strikt zwischen Festangestellten und Leiharbeitern (*n* = 3).
4. Die Studie zielte ausschließlich auf die Pädiatrie (*n* = 1) oder Notaufnahme (*n* = 1).
5. Der Text war ausschließlich ein Editorial bzw. Letter (*n* = 4).
6. Die Studie war nicht auf Englisch oder Deutsch verfügbar (*n* = 1).

Vor der inhaltlichen Auswertung der Studien wurden durch die Qualitätsanalysen nach Hawker weitere vier Volltexte ausgeschlossen. Insgesamt wurden drei Studien im Detail analysiert [19–21]. Es handelte sich um eine quantitative retrospektive Längsschnittstudie, eine Fallstudie und eine qualitative interviewbasierende Arbeit (Tab. 3).

**Table 3:** 

Erstautor und Jahr	Journal	Design	Setting	Ziele	Kernaussagen
Hass Helen (2006) [21]	ICCN	„Husserlian phenomenology (interviews)“	30 Betten Intensiv, Großbritannien, 8 Vollzeit-„Leiharbeiter“	Erfahrungen von hauptamtlichen Agenturpflegekräften, Intensivpflege	Leiharbeiter gehören nicht zum Team, verlieren Fertigkeiten
Bae SH (2015) [19]	JCN	Retrospektive Längsschnittstudie	12 ICU. 144 Monate	Zusammenhang von Leiharbeitern und nosokomialen Infektionen (zentralkatheterassoziierte Blutstrominfektionen und beatmungsassoziierte Pneumonie)	Kein Zusammenhang – aber Arbeitsbedingungen sind wichtig
Shuldham C (2009) [20]	IJNS	„Case study“	2 Kliniken (NHS Trust) in England, 1 ICU	Zusammenhang zwischen Pflegestunden Festangestellter und Leiharbeiter auf Patienten	Schwache Evidenz für Sepsisrate

### Die subjektive Sicht

Hass et al. (2005) haben Interviewaussagen von Vollzeitleiharbeitern auf 30 Intensivstationen ausgewertet [21]. Diese mussten eine Weiterbildung für die Intensivstation und zusätzlich mindestens zwei Jahre Berufserfahrung auf der Intensivstation vorweisen. Aus Sicht der Leiharbeiter geht der Einsatz auf fremden Stationen mit 3 Hauptthemen einher:

1. Festangestellte und Leiharbeiter haben untereinander und miteinander unterschiedliche Arbeitsweisen. Leiharbeiter sind eher vom restlichen Team isoliert.
2. Leiharbeiter berichteten von einer fehlenden Selbstsicherheit. Es bestehen Unsicherheiten, z. B. wo welches Material zu finden sei, insbesondere wenn Checklisten und einheitliche Beschriftungen fehlten. Bei Unsicherheiten wurde beim Stammpersonal ungern nachgefragt.
3. Die eingesetzten Leiharbeiter berichteten von einem Verlust von Fertig- und Fähigkeiten. Sie wurden nicht im selben Umfang wie Festangestellte bei kritischen Patienten eingesetzt. Trotz Einsatz auf der Intensivstation fehlten ihnen regelmäßige Trainings und Wissen in der Ausführung von Notfallmaßnahmen, wie Basic Life Support und Advanced Life Support. Es wurde ihnen eine Eigenständigkeit abgesprochen. Sie durften z. B. nicht selbstständig kreislaufwirksame Medikamente verabreichen oder die Laufraten verändern, auch wenn es notwendig gewesen wäre. Der Patient habe demnach auf die Weisung durch Festangestellte warten müssen. Konkret sei kein Patient zu Schaden gekommen.

Das Limit der Arbeit liegt im Design, was eine Verallgemeinerung auf andere Situationen nicht ermöglicht.

### Einfluss auf das Patientenoutcome – quantitative Sicht

Shuldham et al. (2008) untersuchten in ihrer Fallstudie die Auswirkungen von verschiedenen Personalstrukturen auf pflegesensitive Outcomeparameter durch verschiedene Personalstrukturen [20]. Hierzu wurden in zwei Kliniken Daten retrospektiv aus 12 Monaten mittels Regressionsanalyse betrachtet. Die getrennte Auswertung der Einsatzzeiten (Pflegezeit in Stunden pro Patient) zwischen Festangestellten und Leiharbeitnehmern ergab keine Hinweise auf eine veränderte Inzidenz für Druckgeschwüre, Patientenstürze, Blutungen, Lungenentzündung, Schock und/oder Thrombose. Es wurden nur geringfügige Tendenzen für einen Rückgang der Sepsisrate berichtet, sobald weniger Leiharbeiter (in Stunden/Patient) auf der Intensivstation eingesetzt wurden (IRR = 0,983, 95 %-CI = 0,972–0,995, *p* = 0,006; [20]). Kritisch zu sehen ist die fehlende Powerkalkulation. Ferner gab es eine Datenmischung zwischen Festangestellten und hausinternen sog. „Poolmitarbeitern“. Diese sind Festangestellte in den untersuchten Kliniken. Inwieweit diese regelmäßig auf den untersuchten Stationen eingesetzt wurden und/oder mit spezifischen Standards vertraut waren, wurde nicht berichtet. Ebenso war der Analyse nicht zu entnehmen, ob der Einsatz der Leiharbeiter auf eine veränderte Patienten- und/oder Dienstplansituation zurückzuführen ist. Es konnten z. B. keine Aussagen zum Schweregrad der Erkrankung mittels Score oder Indizes gefunden werden. Aus den genannten Outcomeparametern und Settings und analysierten Daten kann kein erheblicher Nachteil für Patienten durch Leiharbeiter nachgewiesen werden.

### Ergebnisse der Längsschnittstudie

Bae et al. (2014) betrachten in einer Längsschnittstudie Daten von 12 Intensivstationen aus 12 Monaten mit dem Fokus auf die ventilatorassoziierte Pneumonie (VAP) und katheterassoziierten Blutstrominfektionen (CABSI; [19]). Bei der getrennten Betrachtung der Einsatzzeit in Stunden/Patient konnte in keiner Analyse oder Subanalyse ein Zusammenhang zwischen einem Anstieg von Infektionen und dem Einsatz von Leiharbeitern hergestellt werden. Kritisch ist hier die Mischung zwischen Festangestellten und Poolmitarbeitern zu nennen. Es wurde nicht der Schweregrad der Patienten berichtet oder der Grund für den Einsatz der Leiharbeiter. Bemerkenswert sind die Ergebnisse einer Subanalyse von Bae et al. bezüglich der Personalstruktur (Betreuungsschlüssel und Qualifikation), Anzahl der Betten und Berücksichtigung der Arbeitsumgebung. Es zeigten sich Hinweise, dass ein Zusammenhang zwischen einer VAP und einer Blutstrominfektion maßgeblich auf die Größe der Intensivstation und Arbeitsumgebung zurückzuführen war. So stieg die Wahrscheinlichkeit für das Auftreten von katheterassoziierten Infektionen um 14,8 % (95 %-CI = 1,032–1,277, *p* = 0,011) je zusätzliches Bett gegenüber den Vergleichsstationen, nach Adjustierung anderer unterschiedlicher Faktoren. Auffällig war, dass ein größerer Qualifikationsmix mit einem stärkeren Auftreten von VAP assoziiert war. Bae et al. betrachteten hierzu die Practice Environment Scale (PES; [25]). Die PES misst das Führungsverhalten und Unterstützung von Pflegekräften durch Leitungskräfte, die Angemessenheit von Personal und Ressourceneinsatz und die kollegialen Beziehungen zwischen Pflegepersonal und Ärzten. Die Ergebnisse zeigten, dass, unabhängig vom Einsatz von Leiharbeitern, eine Verbesserung um einen Punkt auf der PES die Quote für CABSI um 95 % (95 %-CI = 0,005–0,550, *p* = 0,014) und für VAP um 79 % (95 %-CI = 0,049–0,952, *p* = 0,043) sank.

Die Arbeit ist methodisch gut aufgebaut und nachvollziehbar, dennoch ist zu bemerken, dass die Daten auf den verschiedenen Stationen über das Jahr verteilt zu unterschiedlichen Zeitpunkten erhoben wurden und hierdurch Verzerrungen möglich sind. Ebenfalls kritisch anzumerken ist, dass in der analysierten Gruppe der Leiharbeiter sowohl gelernte als auch ungelernte Pflegekräfte zusammengefasst wurden.

## Diskussion und Limitationen

Die Ergebnisse dieser systematischen Übersichtsarbeit zeigten keinen Zusammenhang zwischen dem Einsatz von Leiharbeitern auf Intensiv- und Überwachungsstationen und einem damit einhergehenden Einfluss auf Mortalität, Liegedauer, Hygienefehler, Infektionen, Dekubitalulzera, körperliche Fixierungen, Pflegezeit, Herz-Kreislauf-Ereignisse sowie allgemeine Patientengefährdungen und Patientensicherheit auf. Angaben, die für eine Normalstation vorliegen und besagen, dass Tage mit mehr als 1,5 h/Patient versorgt durch Zeitarbeiter und/oder 0,5 h/Patient durch ungelernte Hilfskräfte mit einer größeren Wahrscheinlichkeit zu versterben einhergehen [14], konnten für die Intensivstationen nicht bestätigt werden. Der von Hass et al. beschriebene Verlust von Fertigkeiten beruhte auf subjektivem Empfinden. Ein direkter Einfluss durch den unstetigen Einsatz einzelner Personen auf den jeweiligen Stationen ist nur sehr schwer quantitativ messbar. Hierfür würden große Populationsgruppen über einen längeren Zeitraum begleitet und ausgewertet werden müssen.

In einer Metaanalyse von Driscoll et al. (2018) wird beschrieben, dass der Einfluss von Betreuungsschlüsseln auf sogenannte pflegesensitive Outcomeparameter wie Infektionen, Sepsis, Mortalität, Fixierungen und Dekubitalulzera besteht [12]. Allerdings wurde auch hier schon erwähnt, dass es allenfalls schwache Hinweise gibt, dass diese Ergebnisse auf Intensivstationen übertragbar sind [12]. Vermutlich ist dies darauf zurückzuführen, dass auf Intensivstationen von vornherein eine spezialisierte Ausbildung notwendig ist und ein besserer Betreuungsschlüssel gegenüber der Normalstation besteht. Nachfolgende Arbeiten könnten den Einfluss von spezialisierter (Fach‑)Ausbildung ebenso betrachten wie den Einfluss der engen und multiprofessionellen Zusammenarbeit auf der Intensivstation.

Der von Shuldham et al. aufgezeigte, wenn auch als schwach bezeichnete Hinweis auf eine niedrige Sepsisrate durch mehr Betreuungszeit von Festangestellten konnte nicht bestätigt werden. Auffällig war, dass „Poolmitarbeiter“ zu den Festangestellten gezählt wurden. Sollte es einen positiven Einfluss haben, in einem festen Team mit festen Abläufen und Standards zu arbeiten, sollten diese „temporär“ eingesetzten Mitarbeiter entsprechend anders erfasst und analysiert werden. Generell fehlte in allen untersuchten Arbeiten eine Analyse, die zwischen Ursache und Wirkung der Leiharbeiter unterscheidet und den Schweregrad der Patientenerkrankungen erfasste.

Eine Schwäche dieser Übersichtsarbeit besteht in der geringen Übertragbarkeit der Ergebnisse auf einzelne Länder. Qualifikation und Strukturen der Pflege sind unterschiedlich. Eine statistische Gesamtanalyse der Daten war aufgrund der Heterogenität und geringen Menge der gefunden Daten nicht möglich. Eine Aussage über ein Disseminationsbias ist ebenfalls nicht möglich. Ein weiteres Limit ist die, entgegen den PRISMA-Kriterien, fehlende Qualitätsbeurteilung der eingeschlossenen Arbeiten durch einen zweiten unabhängigen Gutachter.

## Schlussfolgerungen

Obwohl kein Zusammenhang zwischen einem veränderten Patientenoutcome und dem Einsatz von Leiharbeitern identifiziert wurde, sollte deren Einsatz schon aus Kostengründen nicht unreflektiert erfolgen. Generell fehlen Analysen, die zwischen Ursache und Wirkung von Leiharbeitern unterscheiden. Es ist weiterhin unklar, ob die Patienten- und/oder Dienstplansituation den Einsatz von Leiharbeitern notwendig macht. In der Bewertung von Outcomeparametern scheint es auch eine Diskrepanz zwischen subjektiven Wahrnehmungen und messbaren Ergebnissen zu geben. Eine Schwierigkeit besteht darin, ein pflegesensitives Outcome zu messen bzw. als solches zu benennen. Das Outcome des Patienten ist multifaktoriell und kann sicher nicht auf eine Profession allein heruntergerechnet werden. Keine der Arbeiten berichtete, wie der Qualifikationsnachweis der eingesetzten Leiharbeiter erfolgt oder die Einarbeitung in neue Bereiche gewährleistet wird. Ferner fand eine Durchmischung mit sog. Poolmitarbeitern und Festangestellten in der Datenanalyse statt. Dies sollte getrennt erfolgen, um einen möglichen Vorteil von festem „Stammpersonal“ zu finden. Im Vergleich zur Normalstation ist auf der Intensivstation ein anderer Besetzungsschlüssel im ärztlichen und pflegerischen Dienst zu finden. Es ist denkbar, dass persönliche Unsicherheiten der Leiharbeiter in Bezug auf die Arbeitsumgebung und Arbeitsabläufe so eher kompensiert werden. Weiter ist denkbar, dass unmittelbar mehrere Ansprechpartner für Fragen zu einem bestimmten Vorgehen vorhanden sind. Drei Dinge beeinflussen hingegen stark das Patientenoutcome. Der jeweilige Betreuungsschlüssel, die Qualifikation und die Arbeitsbedingungen. Letztere führen möglicherweise dazu, dass Mitarbeiter eher in die Leiharbeit wechseln. Dies könnte auf eine andere Bezahlung und eine flexiblere Arbeitsgestaltung zurückzuführen sein. Es bedarf weiterer Studien, um herauszufinden, was ein neuralgisches Verhältnis von Festangestellten zu Leiharbeitern auf der Intensivstation ist und welcher messbare Einfluss auf den Patienten durch den Einsatz entsteht. Dabei könnten Fertig- und Fähigkeiten (z. B. Delirerkennung oder Rate der Frühmobilisation) von Festangestellten und Leiharbeitern direkt verglichen werden.

## Fazit für die Praxis

- Der Einsatz von Leiharbeitern sollte auch auf Intensivstationen reflektiert erfolgen.
- Subjektive Sichtweisen und damit Auswirkungen auf die Zusammenarbeit sollten verbalisiert werden.
- Der Einsatz setzt eine Qualitätssicherung und Einarbeitung voraus.
- Checklisten und standardisierte Abläufe können den Einsatz hilfreich unterstützen.
- Möglichweise gibt es ein neuralgisches Verhältnis von Festangestellten zu Leiharbeitern.
- In jedem Fall scheinen die Arbeitsbedingungen maßgeblich das Outcome zu beeinflussen.

## Caption Electronic Supplementary Material





## Footnotes

1 Artikel im Druck, endgültige Zitationsdetails müssen noch hinzugefügt werden und der Text kann sich vor der endgültigen Veröffentlichung noch ändern.
