# Supplementary material for: Agency work in intensive care: Impact of temporary contract work on patient care in intermediate care and intensive care units
Source: Med Klin Intensivmed Notfmed. 2020 Oct 21;117(1):16–23. [Article in German] doi: 10.1007/s00063-020-00753-5 (PMC8782819; doi:10.1007/s00063-020-00753-5)
Supplement: Supplementary file 1 [file 63_2020_753_MOESM1_ESM.pdf]

Zusatzmaterial – Suchstrategie

Die online Suche wurde teilweise über einen VPN Tunnel der HAW-Hamburg durchgeführt, um auch auf kostenpflichtige Bereiche der Datenbanken zugreifen zu können. Die Datenbanken wurden zwischen Dezember 2019 und Ende März 2020 systematisch mit den angegeben Variablen durchsucht. Eine Überprüfung auf neue Einträge erfolgte zuletzt am 16. April 2020.

**Abhängige variable** - untereinander verbunden mit "OR"		**Unabhängige variable** - untereinander verbunden mit "OR"		**Bereichseingrenzung** - untereinander verbunden mit "OR"
“agency work”		Sideeffect		
“agency work”		"patient outcome"		
“Agency worker”		"patient outcomes"		
“contract work”		Restraint		
“Temp Staff”		“physical restraints”		
"Temporary Staff*"		"pressure ulcers"		
"temporary staffing"		“nursing time”		
"temporary nursing staff"		"nursing hours”		„ICU“
"temporary nursing"		Hygiene		„critical care“
"agency nursing"	AND	Failure-to-rescue	AND	„critical care*“
“Agency Nurse”		"cardiac events"		intensive care unit
“Agency staff”		cardiac events		
“freelancer”		"patient safety"		
"temporary agency work*"		"adverse events"		
“Contract worker”		Infections		
“Travel Nurse”		“Length of stay”		
"self-employed nurse"		"Health provision"		
“temp nurse”		"Healthcare provision"		
		"Mortality"		

Suche in Livivo

1. Suche “open search” für Leiharbeit und Synonyme = 71.095 Treffer

“agency work” OR “Agency worker” OR “contract work” OR “Temp Staff” OR "Temporary Staff*" OR "temporary staffing" OR "temporary nursing staff" OR "temporary nursing" OR "agency nursing" OR “Agency Nurse” OR “Agency staff” OR “freelancer” OR "temporary agency work*" OR “Contract worker” OR “Travel Nurse” OR "self-employed nurse" OR “temp nurse”

2. Suche “open search” für „Nebenwirkungen und Synonyme“ = 4.666.934 Treffer

Sideeffect OR "patient outcome" OR "patient outcomes" OR Restraint OR “physical restraints” OR "pressure ulcers" OR “nursing time” OR “nursing hours” OR Hygiene OR Failure-to-rescue OR "cardiac events" OR cardiac events OR "patient safety" OR "adverse events" OR Infections OR “Length of stay” OR “length of stay” OR "Health provision" OR "Healthcare provision" OR Mortality

3. Suche, Suche 1 „und“ Suche 2 „open search“ = 4.404.075 Treffer

“agency work” OR “Agency worker” OR “contract work” OR “Temp Staff” OR "Temporary Staff*" OR "temporary staffing" OR "temporary nursing staff" OR "temporary nursing" OR "agency nursing" OR “Agency Nurse” OR “Agency staff” OR “freelancer” OR "temporary agency work*" OR “Contract worker” OR “Travel Nurse” OR "self-employed nurse" OR “temp nurse”) AND Sideeffect OR "patient outcome" OR "patient outcomes" OR Restraint OR “physical restraints” OR "pressure ulcers" OR “nursing time” OR “nursing hours” OR Hygiene OR Failure-to-rescue OR "cardiac events" OR cardiac events OR "patient safety" OR "adverse events" OR Infections OR “Length of stay” OR “length of stay” OR "Health provision" OR "Healthcare provision" OR Mortality

4. Suche eingrenzen auf „critical care“ = 358 Treffer

(Sideeffect OR "patient outcome" OR "patient outcomes" OR Restraint OR “physical restraints” OR "pressure ulcers" OR “nursing time” OR “nursing hours” OR Hygiene OR Failure-to-rescue OR "cardiac events" OR cardiac events OR "patient safety" OR "adverse events" OR Infections OR “Length of stay” OR “length of stay” OR "Health provision" OR "Healthcare provision" OR Mortality) AND (“agency work” OR “Agency worker” OR “contract work” OR “Temp Staff” OR "Temporary Staff*" OR "temporary staffing" OR "temporary nursing staff" OR "temporary nursing" OR "agency nursing" OR “Agency Nurse” OR “Agency staff” OR “freelancer” OR "temporary agency work*" OR “Contract worker” OR “Travel Nurse” OR "self-employed nurse" OR “temp nurse”) AND ((("ICU" OR ("intensive care unit") OR "critical care" OR cirtical care*)))

Trip Medical Database

1. Suche “open search” für Leiharbeit und Synonyme = 825 Treffer

(“agency work” OR “Agency worker” OR “contract work” OR “Temp Staff” OR "Temporary Staff*" OR "temporary staffing" OR "temporary nursing staff" OR "temporary nursing" OR "agency nursing" OR “Agency Nurse” OR “Agency staff” OR “freelancer” OR "temporary agency work*" OR “Contract worker” OR “Travel Nurse” OR "self-employed nurse" OR “temp nurse”)

2. Suche “open search” für „Nebenwirkungen und Synonyme“ = 1095335 Treffer

(Sideeffect OR "patient outcome" OR "patient outcomes" OR Restraint OR “physical restraints” OR "pressure ulcers" OR “nursing time” OR “nursing hours” OR Hygiene OR Failure-to-rescue OR "cardiac events" OR cardiac events OR "patient safety" OR "adverse events" OR Infections OR “Length of stay” OR “length of stay” OR "Health provision" OR "Healthcare provision" OR Mortality)

3. Suche, Suche 1 „und“ Suche 2 „open search“ = 378 Treffer

(Sideeffect OR "patient outcome" OR "patient outcomes" OR Restraint OR “physical restraints” OR "pressure ulcers" OR “nursing time” OR “nursing hours” OR Hygiene OR Failure-to-rescue OR "cardiac events" OR cardiac events OR "patient safety" OR "adverse events" OR Infections OR “Length of stay” OR “length of stay” OR "Health provision" OR "Healthcare provision" OR Mortality) AND (“agency work” OR “Agency worker” OR “contract work” OR “Temp Staff” OR "Temporary Staff*" OR "temporary staffing" OR "temporary nursing staff" OR "temporary nursing" OR "agency nursing" OR “Agency Nurse” OR “Agency staff” OR “freelancer” OR "temporary agency work*" OR “Contract worker” OR “Travel Nurse” OR "self-employed nurse" OR “temp nurse”)

4. Suche eingrenzen auf „critical care“ = 74 Treffer

(Sideeffect OR "patient outcome" OR "patient outcomes" OR Restraint OR “physical restraints” OR "pressure ulcers" OR “nursing time” OR “nursing hours” OR Hygiene OR Failure-to-rescue OR "cardiac events" OR cardiac events OR "patient safety" OR "adverse events" OR Infections OR “Length of stay” OR “length of stay” OR "Health provision" OR "Healthcare provision" OR Mortality) AND (“agency work” OR “Agency worker” OR “contract work” OR “Temp Staff” OR "Temporary Staff*" OR "temporary staffing" OR "temporary nursing staff" OR "temporary nursing" OR "agency nursing" OR “Agency Nurse” OR “Agency staff” OR “freelancer” OR "temporary agency work*" OR “Contract worker” OR “Travel Nurse” OR "self-employed nurse" OR “temp nurse”) AND ("ICU" OR "intensive care unit" OR "critical care)

Medline via PRIME

1. Suche für Leiharbeit und Synonyme = 726 Treffer

(“agency work” OR “Agency worker” OR “contract work” OR “Temp Staff” OR "Temporary Staff*" OR "temporary staffing" OR "temporary nursing staff" OR "temporary nursing" OR "agency nursing" OR “Agency Nurse” OR “Agency staff” OR “freelancer” OR "temporary agency work*" OR “Contract worker” OR “Travel Nurse” OR "self-employed nurse" OR “temp nurse”)
2. Suche „Nebenwirkungen und Synonyme“ = 3366085 Treffer

(Sideeffect OR "patient outcome" OR "patient outcomes" OR Restraint OR “physical restraints” OR "pressure ulcers" OR “nursing time” OR “nursing hours” OR Hygiene OR Failure-to-rescue OR "cardiac events" OR cardiac events OR "patient safety" OR "adverse events" OR Infections OR “Length of stay” OR “length of stay” OR "Health provision" OR "Healthcare provision" OR Mortality)

3. Suche, Suche 1 „und“ Suche 2 „open search“ = 89 Treffer

(Sideeffect OR "patient outcome" OR "patient outcomes" OR Restraint OR “physical restraints” OR "pressure ulcers" OR “nursing time” OR “nursing hours” OR Hygiene OR Failure-to-rescue OR "cardiac events" OR cardiac events OR "patient safety" OR "adverse events" OR Infections OR “Length of stay” OR “length of stay” OR "Health provision" OR "Healthcare provision" OR Mortality) AND (“agency work” OR “Agency worker” OR “contract work” OR “Temp Staff” OR "Temporary Staff*" OR "temporary staffing" OR "temporary nursing staff" OR "temporary nursing" OR "agency nursing" OR “Agency Nurse” OR “Agency staff” OR “freelancer” OR "temporary agency work*" OR “Contract worker” OR “Travel Nurse” OR "self-employed nurse" OR “temp nurse”)

4. Suche eingrenzen auf „critical care“ = 11 Treffer

(Sideeffect OR "patient outcome" OR "patient outcomes" OR Restraint OR “physical restraints” OR "pressure ulcers" OR “nursing time” OR “nursing hours” OR Hygiene OR Failure-to-rescue OR "cardiac events" OR cardiac events OR "patient safety" OR "adverse events" OR Infections OR “Length of stay” OR “length of stay” OR "Health provision" OR "Healthcare provision" OR Mortality) AND (“agency work” OR “Agency worker” OR “contract work” OR “Temp Staff” OR "Temporary Staff*" OR "temporary staffing" OR "temporary nursing staff" OR "temporary nursing" OR "agency nursing" OR “Agency Nurse” OR “Agency staff” OR “freelancer” OR "temporary agency work*" OR “Contract worker” OR “Travel Nurse” OR "self-employed nurse" OR “temp nurse”) AND ("ICU" OR "intensive care unit" OR "critical care)

Wiley

1. Suche für Leiharbeit und Synonyme = 8285 Treffer

(“agency work” OR “Agency worker” OR “contract work” OR “Temp Staff” OR "Temporary Staff*" OR "temporary staffing" OR "temporary nursing staff" OR "temporary nursing" OR "agency nursing" OR “Agency Nurse” OR “Agency staff” OR “freelancer” OR "temporary agency work*" OR “Contract worker” OR “Travel Nurse” OR "self-employed nurse" OR “temp nurse”)

2. Suche „Nebenwirkungen und Synonyme“ = 482501 Treffer

(Sideeffect OR "patient outcome" OR "patient outcomes" OR Restraint OR “physical restraints” OR "pressure ulcers" OR “nursing time” OR “nursing hours” OR Hygiene OR Failure-to-rescue OR "cardiac events" OR cardiac events OR "patient safety" OR "adverse events" OR Infections OR “Length of stay” OR “length of stay” OR "Health provision" OR "Healthcare provision" OR Mortality)

3. Suche, Suche 1 „und“ Suche = 1083 Treffer

(Sideeffect OR "patient outcome" OR "patient outcomes" OR Restraint OR “physical restraints” OR "pressure ulcers" OR “nursing time” OR “nursing hours” OR Hygiene OR Failure-to-rescue OR "cardiac events" OR cardiac events OR "patient safety" OR "adverse events" OR Infections OR “Length of stay” OR “length of stay” OR "Health provision" OR "Healthcare provision" OR Mortality) AND (“agency work” OR “Agency worker” OR “contract work” OR “Temp Staff” OR "Temporary Staff*" OR "temporary staffing" OR "temporary nursing staff" OR "temporary nursing" OR "agency nursing" OR “Agency Nurse” OR “Agency staff” OR “freelancer” OR "temporary agency work*" OR “Contract worker” OR “Travel Nurse” OR "self-employed nurse" OR “temp nurse”)

4. Suche eingrenzen auf „critical care“ **= 103 Treffer**

(Sideeffect OR "patient outcome" OR "patient outcomes" OR Restraint OR “physical restraints” OR "pressure ulcers" OR “nursing time” OR “nursing hours” OR Hygiene OR Failure-to-rescue OR "cardiac events" OR cardiac events OR "patient safety" OR "adverse events" OR Infections OR “Length of stay” OR “length of stay” OR "Health provision" OR "Healthcare provision" OR Mortality) AND (“agency work” OR “Agency worker” OR “contract work” OR “Temp Staff” OR "Temporary Staff*" OR "temporary staffing" OR "temporary nursing staff" OR "temporary nursing" OR "agency nursing" OR “Agency Nurse” OR “Agency staff” OR “freelancer” OR "temporary agency work*" OR “Contract worker” OR “Travel Nurse” OR "self-employed nurse" OR “temp nurse”) AND ("ICU" OR "intensive care unit" OR "critical care)

Chochrane (all text)

1. Suche für Leiharbeit und Synonyme = 48 Reviews, 7 Chochrane Protocols and 54 Trials

“agency work” OR “Agency worker” OR “contract work” OR “Temp Staff” OR "Temporary Staff*" OR "temporary staffing" OR "temporary nursing staff" OR "temporary nursing" OR "agency nursing" OR “Agency Nurse” OR “Agency staff” OR “freelancer” OR "temporary agency work*" OR “Contract worker” OR “Travel Nurse” OR "self-employed nurse" OR “temp nurse”

2. Suche „Nebenwirkungen und Synonyme“ = 7020 Reviews, 1472 Chochrane Protocols and 243644 Trials 43 Editorials 25 Special collections 1580 Clinical Answer

Sideeffect OR "patient outcome" OR "patient outcomes" OR Restraint OR “physical restraints” OR "pressure ulcers" OR “nursing time” OR “nursing hours” OR Hygiene OR Failure-to-rescue OR "cardiac events" OR cardiac events OR "patient safety" OR "adverse events" OR Infections OR “Length of stay” OR “length of stay” OR "Health provision" OR "Healthcare provision" OR Mortality

3. Suche: Kombination aus Suche 1 und Suche 2

= 42 Reviews, 7 Chochrane Protocols and 5 Trials

4. Suche, Suche 3 erweitert um “ AND "acute care" OR "ICU" OR "intensive care" OR "Intensive Care Unit" = **2 Reviews und 1 Trial**

Sideeffect OR "patient outcome" OR "patient outcomes" OR Restraint OR “physical restraints” OR "pressure ulcers" OR “nursing time” OR “nursing hours” OR Hygiene OR Failure-to-rescue OR "cardiac events" OR cardiac events OR "patient safety" OR "adverse events" OR Infections OR “Length of stay” OR “length of stay” OR "Health provision" OR "Healthcare provision" OR Mortality in All Text AND “agency work” OR “Agency worker” OR “contract work” OR “Temp Staff” OR "Temporary Staff*" OR "temporary staffing" OR "temporary nursing staff" OR "temporary nursing" OR "agency nursing" OR “Agency Nurse” OR “Agency staff” OR “freelancer” OR "temporary agency work*" OR “Contract worker” OR “Travel Nurse” OR "self-employed nurse" OR “temp nurse” in All Text AND "acute care" OR "ICU" OR "intensive care" OR "Intensive Care Unit" in Title Abstract Keyword - (Word variations have been searched)

CINAHL

(Oberfläche - EBSCOhost Research Databases - Datenbank - CINAHL complete)

1. Suche für Leiharbeit und Synonyme = 1259 Treffer

“agency work” OR “Agency worker” OR “contract work” OR “Temp Staff” OR "Temporary Staff*" OR "temporary staffing" OR "temporary nursing staff" OR "temporary nursing" OR "agency nursing" OR “Agency Nurse” OR “Agency staff” OR “freelancer” OR "temporary agency work*" OR “Contract worker” OR “Travel Nurse” OR "self-employed nurse" OR “temp nurse”

2. Suche „Nebenwirkungen und Synonyme“ = 664624 Treffer

Sideeffect OR "patient outcome" OR "patient outcomes" OR Restraint OR “physical restraints” OR "pressure ulcers" OR “nursing time” OR “nursing hours” OR Hygiene OR Failure-to-rescue OR "cardiac events" OR cardiac events OR "patient safety" OR "adverse events" OR Infections OR “Length of stay” OR “length of stay” OR "Health provision" OR "Healthcare provision" OR Mortality

3. Suche, Suche 1 „und“ Suche = 1083 Treffer

4. Suche eingrenzen auf „critical care“ **= 60 Treffer**

Suchlauf-Alert: "TX (Sideeffect OR "patient outcome" OR "patient outcomes" OR Restraint OR “physical restraints” OR "pressure ulcers" OR “nursing time” OR “nursing hours” OR Hygiene OR Failure-to-rescue OR "cardiac events" OR cardiac events OR "patient safety" OR "adverse events" OR Infections OR “Length of stay” OR “length of stay” OR "Health provision" OR "Healthcare provision" OR Mortality) AND TX (“agency work” OR “Agency worker” OR “contract work” OR “Temp Staff” OR "Temporary Staff*" OR "temporary staffing" OR "temporary nursing staff" OR "temporary nursing" OR "agency nursing" OR “Agency Nurse” OR “Agency staff” OR “freelancer” OR "temporary agency work*" OR “Contract worker” OR “Travel Nurse” OR "self-employed nurse" OR “temp nurse”) AND TX ("acute care" OR "ICU" OR "intensive care" OR "Intensive Care Unit")

PubMed

1. Suche für Leiharbeit und Synonyme = 801 Treffer

“agency work” OR “Agency worker” OR “contract work” OR “Temp Staff” OR "Temporary Staff*" OR "temporary staffing" OR "temporary nursing staff" OR "temporary nursing" OR "agency nursing" OR “Agency Nurse” OR “Agency staff” OR “freelancer” OR "temporary agency work*" OR “Contract worker” OR “Travel Nurse” OR "self-employed nurse" OR “temp nurse”

2. Suche „Nebenwirkungen und Synonyme“ = 4.861.137 Treffer

“side effect” or "patient outcome" or "patient outcomes" or restraint or "physical restraints" or "pressure ulcers" or "nursing time" or "nursing hours" or hygiene or failure-to-rescue or "cardiac events" or cardiac events or "patient safety" or "adverse events" or infections or "length of stay" or "length of stay" or "health provision" or "healthcare provision" or Mortality

3. Suche, Suche 1 „und“ Suche = 115 Treffer

((((((((((((((("agency work"[All Fields] OR "Agency worker"[All Fields]) OR "contract work"[All Fields]) OR "Temp Staff"[All Fields]) OR "temporary staff*"[All Fields]) OR "temporary staffing"[All Fields]) OR "temporary nursing staff"[All Fields]) OR "temporary nursing"[All Fields]) OR "agency nursing"[All Fields]) OR "Agency Nurse"[All Fields]) OR "Agency staff"[All Fields]) OR "freelancer"[All Fields]) OR "temporary agency work*"[All Fields]) OR "Contract worker"[All Fields]) OR "Travel Nurse"[All Fields]) OR "self-employed nurse"[All Fields]) OR ("temp"[All Fields] AND ((((((((((("nurse s"[All Fields] OR "nurses"[MeSH Terms]) OR "nurses"[All Fields]) OR "nurse"[All Fields]) OR "nurses s"[All Fields]) OR "nursing"[MeSH Terms]) OR "nursing"[All Fields]) OR "nursings"[All Fields]) OR "nursing"[MeSH Subheading]) OR "breast feeding"[MeSH Terms]) OR ("breast"[All Fields] AND "feeding"[All Fields])) OR "breast feeding"[All Fields]) OR "nursing s"[All Fields]))) AND (((((((((((((((((("side"[All Fields] AND (((((((("effect"[All Fields] OR "effecting"[All Fields]) OR "effective"[All Fields]) OR "effectively"[All Fields]) OR "effectiveness"[All Fields]) OR "effectivenesses"[All Fields]) OR "effectives"[All Fields]) OR "effectivities"[All Fields]) OR "effectivity"[All Fields]) OR "effects"[All Fields])) OR "patient outcome"[All Fields]) OR "patient outcomes"[All Fields]) OR (((("restraint, physical"[MeSH Terms] OR ("restraint"[All Fields] AND "physical"[All Fields])) OR "physical restraint"[All Fields]) OR "restraint"[All Fields]) OR "restraints"[All Fields])) OR "physical restraints"[All Fields]) OR "pressure ulcers"[All Fields]) OR "nursing time"[All Fields]) OR "nursing hours"[All Fields]) OR (((((("hygiene"[MeSH Terms] OR "hygiene"[All Fields]) OR "hygienic"[All Fields]) OR "hygienical"[All Fields]) OR "hygienically"[All Fields]) OR "hygienics"[All Fields]) OR "hygienization"[All Fields])) OR "failure-to-rescue"[All Fields]) OR "cardiac events"[All Fields]) OR (((("cardiacs"[All Fields] OR "heart"[MeSH Terms]) OR "heart"[All

Fields]) OR "cardiac"[All Fields]) AND (("event"[All Fields] OR "event s"[All Fields]) OR "events"[All Fields]))) OR "patient safety"[All Fields]) OR "adverse events"[All Fields]) OR ((((((((((((((((((("infect"[All Fields] OR "infectability"[All Fields]) OR "infectable"[All Fields]) OR "infectant"[All Fields]) OR "infectants"[All Fields]) OR "infected"[All Fields]) OR "infecteds"[All Fields]) OR "infectibility"[All Fields]) OR "infectible"[All Fields]) OR "infecting"[All Fields]) OR "infection s"[All Fields]) OR "infections"[MeSH Terms]) OR "infections"[All Fields]) OR "infection"[All Fields]) OR "infective"[All Fields]) OR "infectiveness"[All Fields]) OR "infectives"[All Fields]) OR "infectivities"[All Fields]) OR "infects"[All Fields]) OR "pathogenicity"[MeSH Subheading]) OR "pathogenicity"[All Fields]) OR "infectivity"[All Fields])) OR "length of stay"[All Fields]) OR "length of stay"[All Fields]) OR "health provision"[All Fields]) OR "healthcare provision"[All Fields] OR Mortality [All Fields])

4. Suche eingrenzen auf „critical care“ = **18 Treffer**

(((((((((((((((((("Sideeffect"[All Fields] OR "patient outcome"[All Fields]) OR "patient outcomes"[All Fields]) OR (((("restraint, physical"[MeSH Terms] OR ("restraint"[All Fields] AND "physical"[All Fields])) OR "physical restraint"[All Fields]) OR "restraint"[All Fields]) OR "restraints"[All Fields])) OR "physical restraints"[All Fields]) OR "pressure ulcers"[All Fields]) OR "nursing time"[All Fields]) OR "nursing hours"[All Fields]) OR (((((("hygiene"[MeSH Terms] OR "hygiene"[All Fields]) OR "hygienic"[All Fields]) OR "hygienical"[All Fields]) OR "hygienically"[All Fields]) OR "hygienics"[All Fields]) OR "hygienization"[All Fields])) OR "Failure-to-rescue"[All Fields]) OR "cardiac events"[All Fields]) OR (((("cardiacs"[All Fields] OR "heart"[MeSH Terms]) OR "heart"[All Fields]) OR "cardiac"[All Fields]) AND (("event"[All Fields] OR "event s"[All Fields]) OR "events"[All Fields]))) OR "patient safety"[All Fields]) OR "adverse events"[All Fields]) OR ((((((((((((((((((("infect"[All Fields] OR "infectability"[All Fields]) OR "infectable"[All Fields]) OR "infectant"[All Fields]) OR "infectants"[All Fields]) OR "infected"[All Fields]) OR "infecteds"[All Fields]) OR "infectibility"[All Fields]) OR "infectible"[All Fields]) OR "infecting"[All Fields]) OR "infection s"[All Fields]) OR "infections"[MeSH Terms]) OR "infections"[All Fields]) OR "infection"[All Fields]) OR "infective"[All Fields]) OR "infectiveness"[All Fields]) OR "infectives"[All Fields]) OR "infectivities"[All Fields]) OR "infects"[All Fields]) OR "pathogenicity"[MeSH Subheading]) OR "pathogenicity"[All Fields]) OR "infectivity"[All Fields])) OR "Length of stay"[All Fields]) OR "Length of stay"[All Fields]) OR "Health provision"[All Fields]) OR "Healthcare provision"[All Fields] OR Mortality [All Fields]) AND ((((((((((((((("agency work"[All Fields] OR "Agency worker"[All Fields]) OR "contract work"[All Fields]) OR "Temp Staff"[All Fields]) OR "temporary staff*"[All Fields]) OR "temporary staffing"[All Fields]) OR "temporary nursing staff"[All Fields]) OR "temporary nursing"[All Fields]) OR "agency nursing"[All Fields]) OR "Agency Nurse"[All Fields]) OR "Agency staff"[All Fields]) OR "freelancer"[All Fields]) OR "temporary agency work*"[All Fields]) OR "Contract worker"[All Fields]) OR "Travel Nurse"[All Fields]) OR "self-employed

nurse"[All Fields]) OR ("temp"[All Fields] AND ((((((((((("nurse s"[All Fields] OR "nurses"[MeSH Terms]) OR "nurses"[All Fields]) OR "nurse"[All Fields]) OR "nurses s"[All Fields]) OR "nursing"[MeSH Terms]) OR "nursing"[All Fields]) OR "nursings"[All Fields]) OR "nursing"[MeSH Subheading]) OR "breast feeding"[MeSH Terms]) OR ("breast"[All Fields] AND "feeding"[All Fields])) OR "breast feeding"[All Fields]) OR "nursing s"[All Fields])))) AND (("ICU"[All Fields] OR "intensive care unit"[All Fields]) OR (("critical care"[MeSH Terms] OR ("critical"[All Fields] AND "care"[All Fields])) OR "critical care"[All Fields]))
